# Cyclic Voltammetry of C.I. Disperse Orange 62 in an Aqueous Electrolyte

**DOI:** 10.3390/ma16216901

**Published:** 2023-10-27

**Authors:** Thomas Bechtold, Noemí Aguiló-Aguayo, Tung Pham

**Affiliations:** 1 Research Institute of Textile Chemistry and Textile Physics, University of Innsbruck, Hoechsterstrasse 73, 6850 Dornbirn, Austria; noemi.aguilo-aguayo@uibk.ac.at (N.A.-A.); tung.pham@uibk.ac.at (T.P.); 2 Faculty of Chemistry and Pharmacy, University of Innsbruck, Innrain 52, 6020 Innsbruck, Austria

**Keywords:** disperse dye, cathodic reduction, cyclic voltammetry, azo group, decolorization, C.I. disperse orange 62

## Abstract

Disperse dyes are an important group of colorants for dyeing polyester fibers. Approximately 30.000 tons of disperse dyes are released into the waste water annually from spent dyebaths. Therefore, methods for decolorizing such dyes are of general interest. The reductive after-treatment of disperse dyes using reducing agents, such as Na_2_S_2_O_4_, is a widely used process to improve rub fastness through dye reduction. Electrochemical dye reduction could be an alternative process for reductive dye treatment. In this work C.I. Disperse Orange 62 was used as a representative dye to study the direct cathodic reduction of a disperse dye with cyclic voltammetry. As anticipated for dispersed organic matter, relatively low current densities were observed, which strongly depend on the state of dispersion of the dye. The current density was increased by using dispersions prepared through dye precipitation from DMF solution and by the use of N-cetyl-N,N,N,-trimethyl-ammonium bromide as a cationic surfactant. The results demonstrate the successful cathodic reduction of a dispersed organic dye; however, the low solubility of the reaction products in the aqueous electrolyte hinders an efficient cathodic dye reduction.

## 1. Introduction

With an annual production of more than 60 M tons, polyester fibers account for over 50% of the global textile fiber production [1]. Assuming that approximately 50% of the produced polyester fibers are dyed with an average color depth of 2%wt, the estimated annual consumption of disperse dyes amounts to 600,000 tons [2]. Disperse dyes are known for their high degree of color fixation, with dyebath exhaustion ranging from 90% to 99% of the total dye used [3]. During the dyeing process, only an adjustment of the dyebath pH is required, resulting in a relatively low chemical consumption for dyeing. However, due to the extensive quantity of dyed polyester fibers, significant amounts of disperse dye are released into the wastewater with the spent dyebath; e.g., with an average dye fixation rate of 95%, the total mass of disperse dyes in the wastewater accumulates to 30.000 tons per year.

Various methods have been proposed for the decolorization of effluents from textile mills, including physical methods such as membrane filtration and adsorption, biological methods such as anaerobic dye degradation and chemical methods, including oxidation processes [4,5,6]. Additionally, electrochemical processes have been under investigation for dye decolorization [7]. Soluble dye molecules are susceptible to direct anodic or cathodic electron transfer, resulting in rapid electrochemical decolorization through oxidation or reduction [8].

Disperse dyes are typically present in the form of dye particles with low solubility, which means that processes suitable for treating soluble dyes require adaptation [9]. To remove these dyes, physical methods such as adsorption processes can be employed [10]. Dye coagulation and precipitation using aluminum hydroxides have been achieved through electrolysis using dissolving aluminum electrodes [11]. However, adsorption processes and dye precipitation result in substantial amounts of spent adsorbent and precipitate, which then require additional treatment for disposal [4].

The technical robustness of the oxidative processes has resulted in the generation of substantial knowledge regarding the anodic decolorization of textile effluents, including dispersed dye systems. Numerous methods for decolorizing effluents from polyester dyeing through oxidative processes have been proposed in the literature [12,13].

The formation of reactive species using β-PbO_2_ anodes has been used to achieve oxidative decolorization of C.I. Disperse Orange 29 [14]. Similarly, the anodic oxidation of dispersed indigo has been accomplished using boron-doped diamond electrodes [15]. The choice of a supporting electrolyte significantly influences the anodic dye oxidation process. A sulphate-based electrolyte supports the mineralization of the dye through the action of hydroxyl radicals, while a chloride-based electrolyte-mediated electrooxidation leads to the formation of several intermediates [16]. The anodic oxidation of chloride using Ti/RuO_2_ anodes resulted in decolorization and a reduction in chemical oxygen demand (COD) in model dyebaths [17]. However, it is important to note that the anodic oxidation of colorants in the presence of chlorides carries the risk of generating adsorbable halogenated organic compounds (AOX) as unwanted toxic by-products [18]. The anodic generation of oxidative intermediates through Fenton reactions has also been utilized in oxidative dye degradation [16,19,20]. A combination of anaerobic degradation and oxidative electrochemical processes (e.g., Fenton processes) has been proposed as an efficient method for decolorizing and mineralizing colored effluents from textile dyeing [21].

The chemical reduction of dyes containing azo groups with the use of Na_2_S_2_O_4_ and NaOH is a standard procedure for shade correction in reactive dyeing and in the reductive cleaning of polyester dyeing [1]. Consequently, the reductive decolorization of disperse dyes using soluble chemicals is widely used in the after-treatment of polyester dyeing [22].

Reductive processes based on cathodic electron transfer have been utilized to achieve the direct reduction of soluble azo dyes [23]. Additionally, the cathodic generation of reducing intermediates, referred to as mediator systems (e.g., anthraquinoids), has been studied for indirect cathodic azo dye reduction and vat dye reduction in the context of dye reduction and waste water treatment [24]. In these studies, cathodic electron transfer either proceeds directly to the soluble dye molecules or a soluble redox-active chemical is involved as an electron carrier.

However, the direct cathodic decolorization of disperse dyes has not been addressed in scientific studies to date. Disperse dyes for polyester fibers exhibit a certain but low solubility in water, which is a requirement for their application in polyester dyeing from an aqueous dyebath. [1,9]. The minimal dye solubility might allow for cathodic dye reduction, although at very low current densities. Additionally, direct contact between dispersed dye particles and the cathode could facilitate electron transfer.

In this work, we investigate the hypothesis that cathodic electron transfer on finely divided dispersed dyes could be achieved. Consequently, we examine the conditions required for successful cathodic electron transfer to a disperse dye molecule.

**Figure 1:**
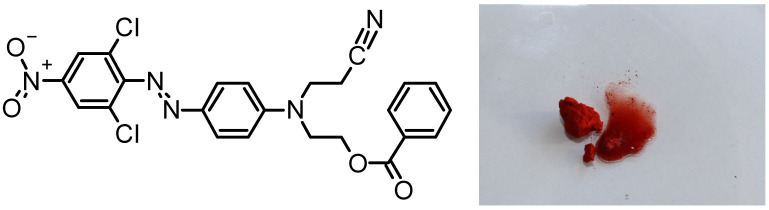
Chemical structure and photograph of C.I. Disperse Orange 62 (DO62). Part of DO62 has been wetted with a drop of water.

C.I. Disperse Orange 62 (Figure 1) was chosen as a representative to investigate the electrochemical behavior of a disperse dye using cyclic voltammetry (CV). In this study, we evaluated the influence of added surfactants and the use of solvent-based electrolytes on the cathodic peak current for dye reduction. Additionally, we conducted a study on indirect cathodic electron transfer through the use of anthraquinone-1,5-disulphonate as a soluble mediator. The results contribute to the scientific foundation for a more comprehensive detailed understanding of cathodic electron transfer to dispersed organic dyes.

## 2. Materials and Methods

### 2.1. Chemicals

The chemicals used for the preparation of the electrolytes used in the voltammetric experiments were analytical-grade chemicals (NaOH, Na_2_SO_4_, LiCl, dimethylformamide (DMF)). 9,10-anthraquinone-1,5-disulphonic acid (AQDS) hydrate, 95%, was used as received (Aldrich-Chemie D-7924, Steinheim, Germany). The press cake of C.I. Disperse Orange 62 was kindly was provided by the University Wuppertal, Germany. The press cake of the dye was used as no dispersants and auxiliaries are present in this non-finished dye. Sodium-dodecylsulphate, nonylphenol-polyethyleneglycol-ether and N-cetyl-N,N,N,-trimethyl-ammonium bromide (CET) were used as delivered (Merck, Darmstadt, Germany).

### 2.2. Cyclic Voltammetry

CV experiments were performed on a hanging mercury drop electrode (HMDE) (EG&G 264 A potentiostat and a 303A electrode stand, drop size small, 0.96 mm²) equipped with a (Ag/AgCl, 3 M KCl) reference electrode and a platinum wire as a counter electrode. Solutions were degassed for 8 min with argon. The pH of the electrolyte was measured with a potentiometer equipped with a glass electrode. To study the electrochemical properties of the disperse dye without further processing, the press cake was added to the supporting electrolyte without additives. In a second series of voltammograms, the disperse dye was first dissolved in DMF (e.g., 3 g L^−1^), then 0.1 mL of the dye solution was added to 10 mL of the respective supporting electrolyte.

A concentration of 0.2 g L^−1^ surfactant was used in experiments to study the influence of surface-active substances on the CV of the dispersion. The dye was precipitated through the addition of 0.2 mL of a 3 g L^−1^ dye solution in DMF to the aqueous supporting electrolyte (10 g L^−1^ Na_2_SO_4_, 4 g L^−1^ NaOH).

A Pt disk electrode (2 mm diameter, 3.14 mm^2^) and a (Ag/AgCl, 3 M KCl) reference electrode were used for CV experiments with a dye solution in the LiCl/DMF electrolyte. The dye-containing electrolyte was prepared by mixing a DMF solution of DO62 (30.8 g L^−1^) with a 10.6 g L^−1^ LiCl-containing DMF supporting electrolyte. Different concentrations of DO62 in the electrolyte were prepared. The final concentrations of DO62 and LiCl are given in the Supporting Information (Table S1). A list of the experimental conditions is presented in Table 1.

The HMDE electrode was used for CV experiments in the aqueous electrolyte as the mercury electrode allows a wider potential range and offers a fresh electrode surface in every experiment. The Pt electrode was used for solvent-based electrolytes.

## 3. Results and Discussion

### 3.1. Cyclic Voltammetry of DO62 in Dissolved State

**Figure 2:**
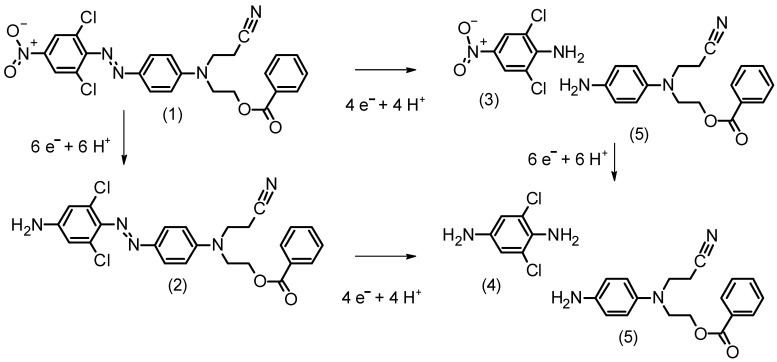
Reaction scheme for the cathodic reduction of reducible groups in DO62; (1) DO62; (2) and (3) partially reduced intermediate compounds; (4) 2,6-dichloro-p-phenylenediamine; (5) N-(2-cyanoethyl-)-N-2-benzoyloxyethyl-)-p-phenylenediamine.

In DO62, both the azo group and the nitro group can undergo cathodic reduction processes [25,26,27]. The reduction of the nitro group leads to the formation of hydroxylamine or amino functionalities, while the reductive cleavage of the azo group results in the formation of two amino groups. In the case of the azo group reduction into amino groups, the chromophoric system of the dye becomes irreversibly destroyed. A simplified reaction scheme depicting possible cathodic reduction reactions is provided in Figure 2.

The reduction of the nitro group in DO62 leads to the formation of the corresponding amino functionality (2), while the reduction of the azo group leads to the cleavage of the azo linkage and the formation of two fragments (3) and (5). The complete reduction of both reducible groups then results in the formation of 2,6-dichloro-p-phenylenediamine (4) and N-(2-cyanoethyl-)-N-(2-benzoyloxyethyl-)-p-phenylenediamine (5). In the literature, the chemical reduction of DO62 with Na_2_S_2_O_4_ has been shown to produce the products (4) and (5), which were identified using mass spectrometry [28]. It is important to note that the reactions presented in Figure 2 have been simplified, as they do not encompass the formation of nitroso and hydroxylamine groups, as well as the formation of hydrazo intermediates.

Disperse dyes have very low solubility in water, so in an aqueous system, DO62 exists in a dispersed form. Even at elevated temperatures, only a very small fraction is in the solution state. For example, for C.I. Disperse Red 1, a maximum concentration of 0.02 M is achieved in water at 80 °C [9].

**Figure 3:**
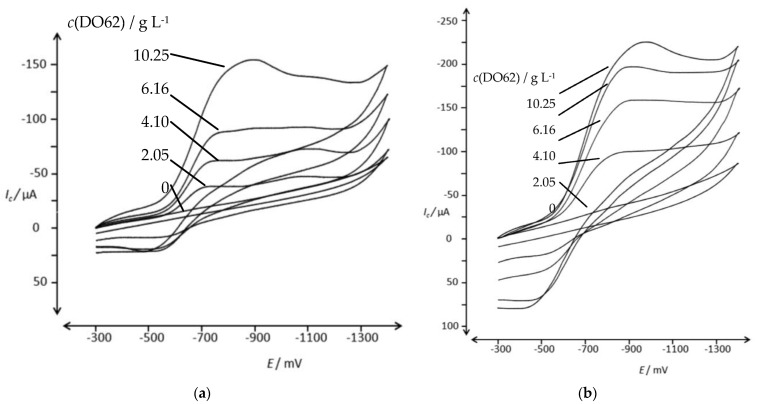
CV of different concentrations of DO62 (0; 2.05; 4.10; 6.16; 10.25 g L^−1^) solution in LiCl/DMF electrolyte; (**a**) 20 mV s^−1^ scan rate and (**b**) 100 mV s^−1^ scan rate on a glassy carbon electrode.

DO62 is readily soluble in DMF. Therefore, in the first set of CV experiments, the cathodic reduction of DO62 was investigated in a DMF solution with LiCl as the supporting electrolyte (Figure 3).

The irreversible dye reduction begins at a cathode potential below −600 mV. Below this potential, the cathodic current increases with dye concentration. The formation of oxidizable products is observed during the reverse scan, occurring above a cathode potential of −600 mV. The cathodic peak current *(I_p_)_c_* falls in the range of −100 µA to −200 µA, with a current density of 3.18 mA cm^−2^ to – 6.37 mA cm^−2^, which is as expected for the reduction of dissolved azo dye molecules.

**Figure 4:**
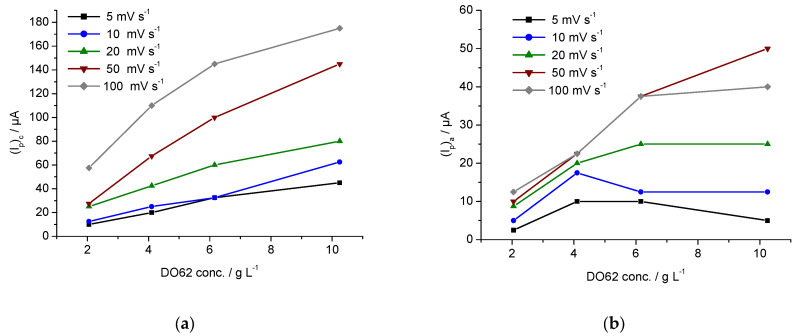
(**a**) Cathodic peak potential *(I_p_)_c_* and (**b**) anodic peak potential *(I_p_)_a_* as function of concentration of DO62 in LiCl/DMF solution for different scan rates (5; 10; 20; 50; 100 mV s^−−1^).

The cathodic and anodic peak currents, *(I_p_)_c_* and *(I_p_)_a_*, are displayed in Figure 4a,b and provided in the Supplementary Information (Table S1). The increase in the cathodic peak current *(I_p_)_c_* is influenced by the dye concentration in the electrolyte. However, at higher scan rates, a non-linear behavior is observed. On the other hand, the anodic peak current *(I_p_)_a_* observed during the reverse scan is much lower, and its increase *(I_p_)_a_* with the scan rate and dye concentration is less pronounced. As indicated in Figure 2, a variety of different oxidizable products with differing reactivity may be present at the end of the cathodic scan. This complexity leads to the observed situation during the anodic scan in the CV.

### 3.2. Cyclic Voltammetry of Dispersed Dyes in Aqueous Electrolyte

**Figure 5:**
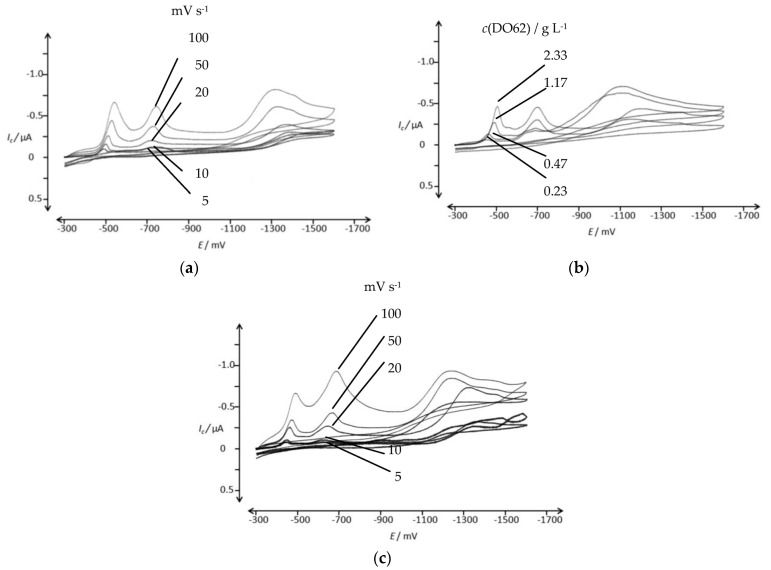
CV of DO62 in aqueous electrolyte in the potential range of −300 mV and −1600 mV; (**a**) 2.31 g L^−1^ DO62 in 10 g L^−1^ Na_2_SO_4_ and 0.6 g L^−1^ NaOH, with pH 12.11 at different scan rates: 5, 10, 20, 50 and 100 mV s^−1^; (**b**) 0.23, 0.47, 1.17 and 2.33 g L^−1^ DO62 in 10 g L^−1^ Na_2_SO_4_ and 0.28 g L^−1^ NaOH, with pH 11.0–11.8, at a scan rate of 50 mV s^−1^; (**c**) 2.02 g L^−1^ DO62 in 10 g L^−1^ Na_2_SO_4_ at different scan rates: 5, 10, 20, 50 and 100 mV s^−1^.

A dispersion of DO62 in an aqueous electrolyte was prepared and studied using CV. The reduction behavior was examined in two alkaline electrolytes: one containing 10 g L^−1^ of Na_2_SO_4_ and 0.6 g L^−1^ of NaOH (Figure 5a), and the other containing 10 g L^−1^ of Na_2_SO_4_ and 0.28 g L^−1^ of NaOH (Figure 5b). Additionally, the reduction behavior was studied in a neutral electrolyte containing (10 g L^−1^ Na_2_SO_4_; Figure 5c). The peak potential and peak current for Figure 5a are provided in the Supplementary Information (Table S2).

Two irreversible reduction peaks are observed in the potential range between −500 mV and −800 mV, and they can be attributed to the reduction of the nitro group and the azo group present in DO62. No reverse peaks were observed. The peak currents for both signals are dependent on the scan rate (Figure 5a) and on the dye concentration (Figure 5b). It is worth noting that the peak currents are relatively low, especially for the relatively high dye concentration used. For instance, at a concentration of 2.31 g L^−1^ DO62 and a scan rate of 50 mV s^−1^, the cathodic peak currents for the two peaks, *(I_p_)_c_*, are 0.53 µA (−550 mV) and 0.28 µA at an *(E_p_)_c_* of −550 mV and −750 mV, respectively. The cathodic peak current densities of 55 µA cm^−2^ and 29 µA cm^−2^ are significantly lower compared to soluble dye systems.

For the cathodic reduction of water-soluble 3,4-dihydroxy-9,10-dioxo-2-anthracene-sulphonate, a cathodic peak current, *(I_p_)_c_*, of 4 µA (0.42 mA cm^−2^) was observed at a scan rate of 50 mV s^−1^ and at a dye concentration of 0.68 g L^−1^ (2 mM). This is nearly ten times higher than what was observed with DO62 in this study [29].

Regarding the particle size in the dry press cake of DO62, the literature reports an average diameter of 2.3 µm. However, in aqueous dispersion, smaller particles tend to combine into larger aggregates. As a result, an average particle diameter of 81.3 µm has been observed in aqueous dispersion [28]. It is highly likely that cathodic reduction occurs for particles that are in direct surface contact with the cathode. In this case, the state of dispersion significantly influences the observed current density.

**Figure 6:**
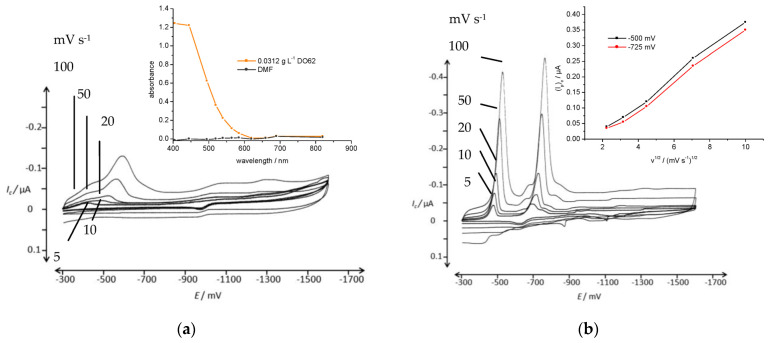
CV of DO62 (0.03 g L^−1^) precipitated from DMF solution in aqueous electrolyte in the potential range of −300 mV and −1600 mV; (**a**) 10 g L^−1^ Na_2_SO_4_ at different scan rates: 5, 10, 20, 50 and 100 mV s^−1^; insert: absorbance of 0.03 g L^−1^ DO62 in DMF; (**b**) 10 g L^−1^ Na_2_SO_4_ and 4 g L^−1^ NaOH at different scan rates: 5, 10, 20, 50 and 100 mV s^−1^; insert: cathodic peak current as a function of the square root of scan rate for both cathodic current peaks.

In a subsequent series of experiments, the disperse dyes were first dissolved in DMF to obtain a solution with a dye concentration of 3 g L^−1^. The solubility of DO62 in DMF was confirmed through photometry. The absence of insoluble dispersed dye particles was demonstrated by the low absorbance of the solution in the wavelength interval ranging from 620 nm to 820 nm (Figure 6).

A volume of 0.1 mL was added to 10 mL of the aqueous supporting electrolyte to precipitate the dye and create a dispersion at a concentration of 0.03 g L^−1^. As anticipated, significantly higher cathodic currents were obtained in the CV of DO62 (Figure 6).

In the CV of the supporting electrolyte with the addition of DMF, no reduction peaks were observed. However, when an alkaline supporting electrolyte was used, two reduction peaks with substantially higher cathodic peak currents, *(I_p_)_c_*, were obtained for DO62. No reverse peaks were observed in the potential range between −300 mV and −1600 mV. The cathodic peak potentials, *(E_p1_)_c_*, and the corresponding cathodic peak currents, *(I_p1_)_c_*, are listed in the Supplementary Information (Table S3).

**Figure 7:**
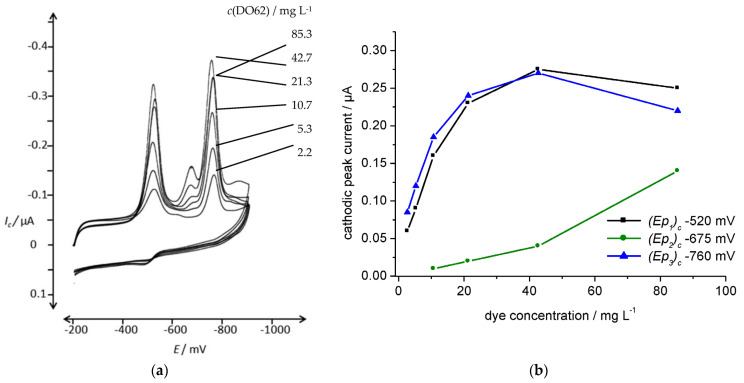
(**a**) CV of different concentrations of DO62, 2.7, 5.3, 10.7, 21.3 and 42.7, 85.3 mg L^−1^ (precipitated from DMF solution); supporting electrolyte, 10 g L^−1^ Na_2_SO_4_ and 4.24 g L^−1^ NaOH; scan rate, 100 mV s^−1^; potential range, from −200 mV and −900 mV; (**b**) cathodic peak current, *(I_p1_)_c_*, at an *(E_p1_)_c_* of −520 mV; *(I_p2_)_c_* at an *(E_p2_)_c_* of −675 mV; and *(I_p3_)_c_* at an *(E_p3_)_c_* of −760 mV, as a function of dye concentration; scan rate, 100 mV s^−1^.

The increased dispersion of DO62 after precipitation from DMF solution into the aqueous electrolyte resulted in higher a cathodic current density during the cathodic scan. In Figure 7, a series of CV scans of freshly precipitated DO62 in the Na_2_SO_4_/NaOH electrolyte with different concentrations of DO62 (ranging from 2.67 g L^−1^ to 85.3 g L^−1^) are presented.

The voltammograms in Figure 7 demonstrate an increase in the cathodic peak currents with increasing dyestuff concentration. However, above a dyestuff concentration of 0.25 mg L^−1^, no further increase in cathodic current is observed. The plateau in cathodic peak current with the increasing concentration of precipitated dye can be explained by a concentration limit. Beyond this limit, the agglomeration of precipitates increases, and thus, there is no further increase in the number of dispersed particles in close contact with the cathode.

At higher dyestuff concentrations, a third reduction peak appeared at *(E_p_)_c_* −680 mV, indicating the complex nature of the cathodic dye reduction (as shown in Figure 2). The cathodic peak potential and peak currents are given in the Supplementary Information (Table S4).

Different surfactants were used to investigate the effect of surface-active substances on the dispersion of the dye and the corresponding cathodic peak currents in the CV experiments. Three different surfactants were employed: sodium-dodecylsulphate (representative for an anionic surfactant), nonylphenol-polyethyleneglycol-ether (non-ionic surfactant) and N-cetyl-N,N,N,-trimethyl-ammonium bromide (CET, cationic surfactant). No significant effects were observed in the presence of sodium-dodecylsulphate or nonylphenol-polyethyleneglycol-ether.

**Figure 8:**
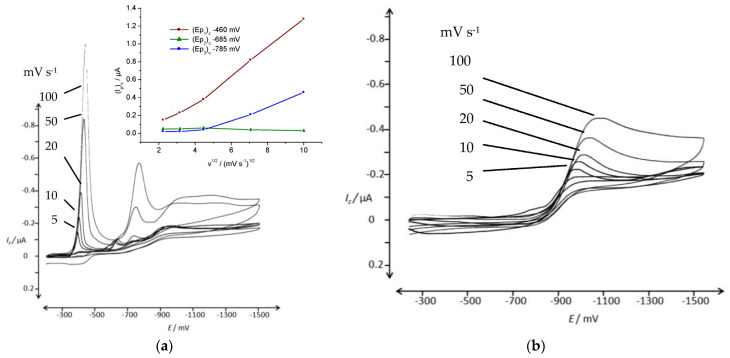
(**a**) CV of DO62 (0.03 g L^−1^) precipitated from DMF solution in aqueous electrolyte in the potential range of −250 mV and −1550 mV in 10 g L^−1^ Na_2_SO_4_ and 4 g L^−1^ NaOH, in the presence of 0.2 g L^−1^ CET at different scan rates: 5, 10, 20, 50 and 100 mV s^−1^; insert: cathodic peak currents as a function of the square root of the scan rate (*v*^1/2^); (**b**) CV of the supporting electrolyte: 0.2 mL DMF solution in 20 mL aqueous electrolyte in 10 g L^−1^ Na_2_SO_4_ and 4 g L^−1^ NaOH, in the presence of 0.2 g L^−1^ CET at different scan rates, 5, 10, 20, 50 and 100 mV s^−1^, in the potential range of −260 mV and −1560 mV.

However, the addition of the cationic surfactant CET resulted in a substantial increase in the cathodic peak currents (as shown in Figure 8). Without a precipitation from a DMF solution, the aqueous dispersions of DO62 in the presence of CET exhibited a lower average particle diameter of 52.0 µm compared to 81.3 µm without the surfactant. The CV of the respective supporting electrolyte is presented in the Figure 8b, and the cathodic peak potential and the peak currents are given in the Supplementary Information.

**Figure 9:**
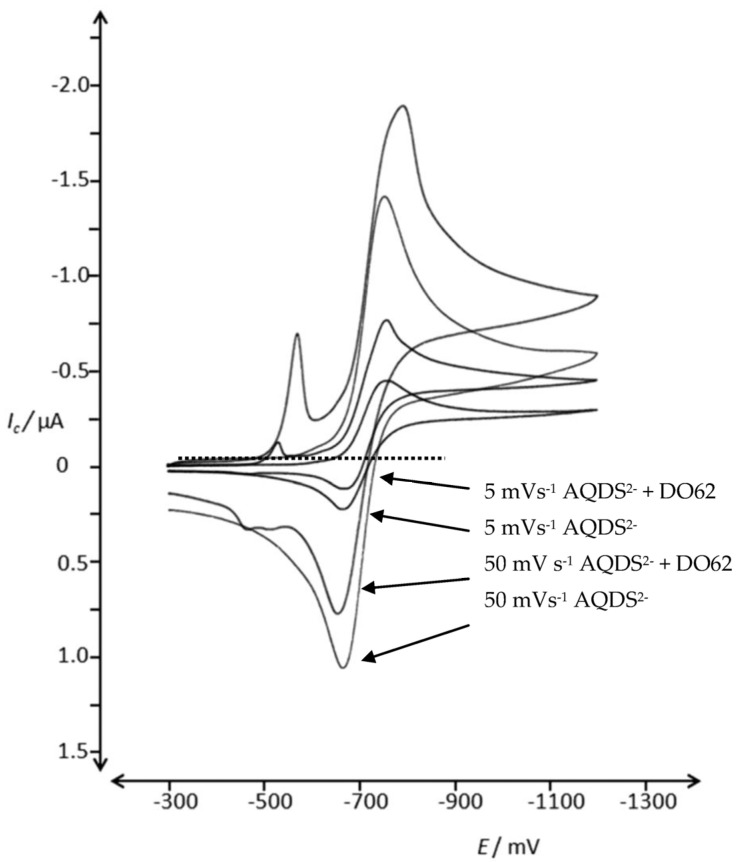
CV of 0.51 mM 9,10-anthraquinone-1,5-disulphonic acid (AQDS) in 10 g L^−1^ Na_2_SO_4_ and 4 g L^−1^ NaOH in the potential range of −300 mV and −1200 mV at scan rates of 5 and 50 mV s^−1^ and in the presence of DO62 (1.46 g L^−1^) precipitated from DMF solution.

In previous studies, soluble reversible redox couples have been successfully used for the indirect cathodic reduction of dispersed vat dyes, including indigo. As an example, for a reversible soluble redox couple, 9,10-anthraquinone-1,5-disulphonic acid (AQDS) was used to facilitate the cathodic reduction of dispersed DO62. In the CV experiments conducted under slightly acidic conditions (pH 5), no electron transfer from reduced AQDS to the dispersed dye was observed (data not provided). However, in an alkaline electrolyte, an electron transfer from cathodically formed AQDS^2-^ to DO62 was observed in the CV experiments (as shown in Figure 9).

The CV of AQDS exhibits the expected shape for a reversible cathodic and anodic electrode reaction. In the presence of DO62, the reduction potential of AQDS^2−^ is sufficient to reduce dispersed DO62 in a heterogeneous reaction. For vat dyes and indigo, the reduction leads to the formation of a soluble leuco dye, resulting in an increase in the cathodic peak current, *(I_p_)_c_*, of AQDS and a reduction of the anodic peak current, *(I_p_)_a_*, of AQDS^2-^ during the reverse scan. Consequently, a distinct catalytic current is observed for these systems.

However, in the case of DO62, the solubility of the follow-up products resulting from an electron transfer from AQDS^2-^ to DO62 in water is low. As a result, the catalytic reaction during the cathodic scan is less pronounced. This leads to a lower anodic peak current, *(I_p_)_a_*, in particular at a low scan rate of 5 mVs^−1^, which is an indication of an electron transfer from AQDS^2−^ to DO62 (as described in Equations (1) and (2)).

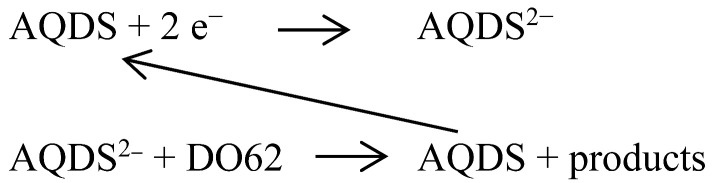
(1)(2)

The CV of AQDS at scan rates from 5 to 100 mVs^−1^ and in the presence of DO62 are shown in the Supplementary Information.

**Figure 10:**
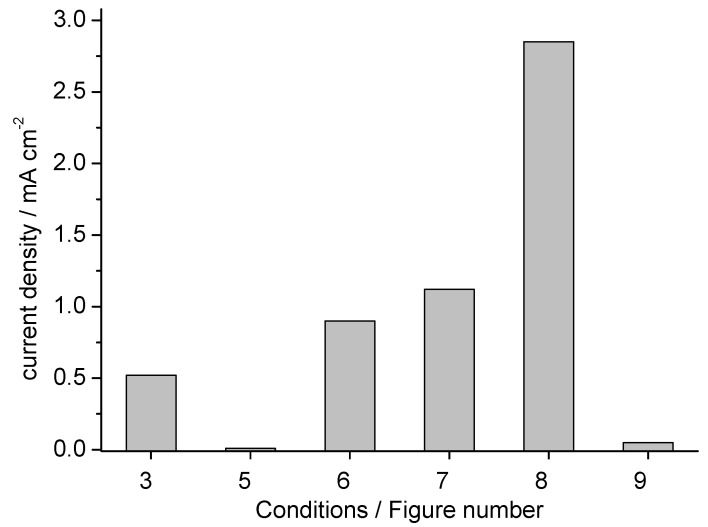
Comparison of cathodic peak current density, *(i_p_)*_c_, for different CV experiments with DO62. Figure (3): LiCl/DMF electrolyte; Figure (5): dispersed press cake; Figures (6) and (7): precipitation from DMF solution; Figure (8): precipitation in presence of CET, Figure (9): precipitation from DMF in presence of AQDS; concentration of DO62 standardized to 1 g L^−1^ at a scan rate of 50 mV s^−1^.

A comparison of the different reduction experiments was conducted by normalizing the respective cathodic peak current density, *(i_p_)*_c_, near *(E_p_)_c_* −500 mV at a scan rate of 50 mV s^−1^ to a reference concentration of 1 g L^−1^ DO62. The results are presented in Figure 10. A substantial increase in the current density is achieved with dispersions of DO62 that have been precipitated from a DMF solution (as shown through CV in Figure 6 and Figure 7). The addition of a cationic surfactant further increases the current density (as observed through CV in Figure 8), while the use of a mediator system did not lead to a substantial increase in current density.

The dispersion of the dye in the electrolyte plays a crucial role in determining the overall current density during dye reduction. Another complication factor arises from the insolubility of the reduction products. In the case of vat dye reduction, the reduced form of the dye, known as the leuco dye, dissolves in an alkaline solution, allowing the dye reduction to continue. However, when reducing DO62, insoluble products are formed. As a result, these reduction products coat the surface of the dispersed particles, hindering further dye reduction.

## 4. Conclusions

The reductive destruction of a dye chromophore by dissolved reducing chemicals is a well-established procedure commonly used for the removal of weakly bound disperse dyes from the surface of polyester fibers. The direct cathodic reduction of C.I. Disperse Orange 62 was successfully achieved after dissolving the dye in a DMF/LiCl electrolyte. Using a Pt cathode, a normalized peak current density of 0.52 mA cm^−2^ was observed for DO62 in the DMF/LiCl solution at a cathode potential below −600 mV and a scan rate of 50 mV s^−1^ (as shown in Figure 10).

In the cathodic reduction of disperse dyes, the electron transfer must occur from the cathode to the dispersed organic phase. Consequently, very low current densities in the order of 10 µA cm^−2^ were calculated for a 1 g L^−1^ dispersion of DO62 press cake in an alkaline aqueous electrolyte. Higher current densities were obtained with freshly precipitated DO62 dispersions and in the presence of the cationic surfactant CET. For these cases, current densities of up to 1.1 mA cm^−2^ and 2.85 mA cm^−2^, respectively, were calculated for a 1 g L^−1^ dispersion of DO62 based on the CV experiments.

The results demonstrate that a direct reduction of dispersed organic pigments can be achieved. However, several factors need to be considered, including the low current density, the solubility of products, and a strong dependency of current density on the experimental conditions such as the presence of auxiliaries. In addition to the heterogeneous electrode reaction, the insolubility of the reaction products represents another limitation for achieving complete dye reduction.

Further investigations could explore cathodic dye reduction at elevated temperatures, which might increase the dye solubility and the solubility of the follow-up products. Alternatively, using acidic conditions during electrolysis could enhance the solubility of the follow-up products by protonating amino functionalities formed during the reduction of the azo group and the nitro groups present in DO62.

The results highlight valuable strategies for the electrochemical reduction of dispersed organic material. Besides the decolorization of dyes in textile waste water, the presented concepts will be of interest for the electrochemical reduction of chemicals in organic synthesis as well as for battery systems that contain dispersed material for increased capacity.

## Figures and Tables

**Table 1:** List of the experimental conditions used in the CV experiments.

DO62 Dissolution Variants	Electrode	Electrolyte	DO62 Concentrationg L^−1^
1—fully dissolved	Pt	LiCl/DMF	0–10.25
2—dispersed, partially dissolved in aqueous electrolyte	HMDE	Na_2_SO_4_/NaOH	0–2.33
3—dispersed, partially dissolved with the presence of surfactants	HMDE	Na_2_SO_4_/NaOH/CET	0, 0.030
4—dispersed, partially dissolved with the presence of a mediator	HMDE	Na_2_SO_4_/NaOH/AQDS	0, 1.46

## Data Availability

The data presented in this study are available on request from the corresponding author.

## Supplementary Materials

The following supporting information can be downloaded at https://www.mdpi.com/article/10.3390/ma16216901/s1: Table S1. Cathodic peak potential, (Ip)c, and anodic peak potential, (Ip)a, of DO62 solutions in LiCl/DMF electrolyte. Table S2. CV of C.I. Disperse Orange 62 in aqueous electrolyte. Table S3. CV of C.I. Disperse Orange 62 (0.03 g L−1) precipitated from DMF solution in aqueous electrolyte. Table S4. Cathodic peak potential (Ep)c and peak current (Ip)c of C.I. Disperse Orange 62 precipitated from DMF solution. Table S5. CV of C.I. Disperse Orange 62 precipitated from DMF solution in aqueous electrolyte in the presence of CET. Figure S1. CV of DO62 in the presence of AQDS.
