# Supplementary material for: Cyclic Voltammetry of C.I. Disperse Orange 62 in an Aqueous Electrolyte
Source: Materials (Basel). 2023 Oct 27;16(21):6901. doi: 10.3390/ma16216901 (PMC10647369; doi:10.3390/ma16216901)
Supplement: Supplementary file 1 [file materials-16-06901-s001.zip › materials-2676883-supplementary.pdf]

Supplementary Information

Cyclic Voltammetry of C.I. Disperse Orange 62 in an Aqueous Electrolyte

Table S1. Composition of DO62 solutions in LiCl/DMF electrolyte used for CV experiments and cathodic peak potential $(I_p)_c$ and anodic peak potential $(I_p)_a$.

Solution	c(DO62)<br>g L^{-1}	c(LiCl)<br>g L^{-1}	Scan rate<br>mV s^{-1}	$(I_p)_c$<br>μA	$(I_p)_a$<br>μA
1	2.05	9.39	5	10	2.5
			10	12.5	5
			20	25	8.75
			50	27.5	10
			100	57.5	12.5
2	4.10	8.72	5	20	10
			10	25	17.5
			20	42.5	20
			50	67.5	22.5
			100	110	22.5
3	6.16	8.05	5	32.5	10
			10	32.5	12.5
			20	60	25
			50	100	37.5
			100	145	37.5
4	10.25	6.71	5	45	5
			10	62.5	12.5
			20	80	25
			50	145	50
			100	175	40

Table S2. Cathodic peak potential $(E_p)_c$ and peak current $(I_p)_c$ of C.I. Disperse Orange 62 (2.31 g L^{-1}) in aqueous electrolyte in the potential range from -300 mV and -1600 mV, scan rates 5, 10, 20, 50, 100 mV s^{-1}; electrolyte:10 g L^{-1} Na_2SO_4, 0.6 g L^{-1} NaOH.

v<br>mV s^{-1}	$(E_{p1})_c$<br>mV	$(I_{p1})_c$<br>µA	$(E_{p2})_c$<br>mV	$(I_{p2})_c$<br>µA
5	-490	0.075	(-750)	0.05
10	-500	0.125	-720	0.05
20	-515	0.2	-720	0.075
50	-530	0.325	-730	0.15
100	-550	0.5375	-750	0.275

Table S3. Cathodic peak potential $(E_p)_c$ and peak current $(I_p)_c$ of C.I. Disperse Orange 62 (0.03 g L^{-1}) precipitated from DMF solution in the potential range from -300 mV and -1600 mV; electrolyte 10 g L^{-1} Na_2SO_4, 4 g L^{-1} NaOH; scan rates 5, 10, 20, 50, 100 mV s^{-1}.

v<br>mV s^{-1}	$(E_{p1})_c$<br>mV	$(I_{p1})_c$<br>µA	$(E_{p2})_c$<br>mV	$(I_{p2})_c$<br>µA
5	-475	0.04	-690	0.035
10	-480	0.07	-710	0.055
20	-490	0.12	-725	0.105
50	-510	0.26	-745	0.235
100	-525	0.375	-760	0.35

Table S4. Cathodic peak potential $(E_p)_c$ and peak current $(I_p)_c$ of C.I. Disperse Orange 62 (2.7, 5.3, 10.7, 21.3, 42.7, 85.3, mg L^{-1}) precipitated from DMF solution, in 10 g L^{-1} Na_2SO_4, 4.24 g L^{-1} NaOH at 100 mV s^{-1}

c(DO62)<br>mg L^{-1}	$(E_{p1})_c$<br>mV	$(I_{p1})_c$<br>µA	$(E_{p2})_c$<br>mV	$(I_{p2})_c$<br>µA	$(E_{p3})_c$<br>mV	$(I_{p3})_c$<br>µA
2.7	-520	0.060	(-400)	(0.005)	-760	0.085
5.3	-520	0.09	-	-	-750	0.12
10.7	-520	0.16	-680	0.01	-760	0.185
21.3	-515	0.23	-670	0.02	-760	0.24
42.7	-520	0.275	-670	0.04	-750	0.27
85.3	-525	0.25	-675	0.14	-760	0.22

Table S5. Cathodic peak potential $(E_p)_c$ and peak current $(I_p)_c$ of C.I. Disperse Orange 62 (0.03 g L^{-1}) precipitated from DMF solution, in the potential range from -250 mV and -1550 mV in 10 g L^{-1} Na_2SO_4, 4 g L^{-1} NaOH, in presence of 0.2 g L^{-1} N-cetyl-N,N,N,-trimethyl-ammonium bromide; scan rates 5, 10, 20, 50, 100 mV s^{-1}.

v mV s^{-1}	$(E_{p1})_c$ mV	$(I_{p1})_c$ µA	$(E_{p2})_c$ mV	$(I_{p2})_c$ µA	$(E_{p3})_c$ mV	$(I_{p3})_c$ µA
5	-440	0.15	-665	0.05	-770	0.02
10	-450	0.23	-675	0.05	-780	0.02
20	-460	0.38	-685	0.06	-785	0.04
50	-485	0.82	-690	0.04	-800	0.21
100	-495	1.28	-700	0.03	-820	0.46

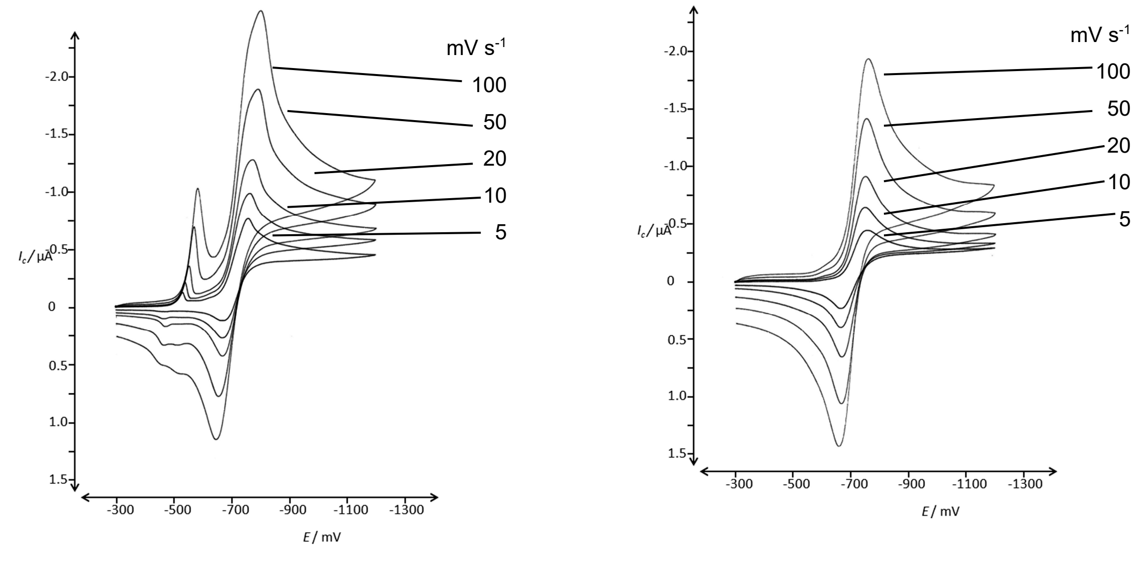

b

a

Figure S1. a) Cyclic voltammetry of DO62 (1.46 g L^{-1}) precipitated from DMF solution in aqueous electrolyte in the potential range from -300 mV and -1200 mV in 10 g L^{-1} Na_2SO_4, 4 g L^{-1} NaOH, in presence of 0.51 mM 9,10- anthraquinone-1,5-disulphonic acid (AQDS) at different scan rates 5, 10, 20, 50, 100 mV s^{-1}; b) 0.51 mM 9,10-anthraquinone-1,5-disulphonic acid (AQDS) in the same ground electrolyte at different scan rates 5, 10, 20, 50, 100 mV s^{-1}.
